# Supplementary material for: Cathepsin B p.Gly284Val Variant in Parkinson’s Disease Pathogenesis
Source: Int J Mol Sci. 2022 Jun 25;23(13):7086. doi: 10.3390/ijms23137086 (PMC9266886; doi:10.3390/ijms23137086)
Supplement: Supplementary file 1 [file ijms-23-07086-s001.zip › Supplementary Material.pdf]

Supplementary Table S1. All variants shared in both affected siblings with MAF <0.01 (NonFinnish European, gnomAD).

Supplementary Table S2. The CTSB variants (MAF <0.01, Non-Finnish European, gnomAD) reported in Mayo Clinic Florida familial Parkinson's disease cohort and Biobank control cohort.

Supplementary Video S1. The video of neurological examination of proband which revealed severe bradykinesia and rest tremor in Med-OFF, Stim-OFF and relief of the symptoms in Med-ON, Stim-ON. In affected brother right hand rest tremor and bradykinesia was noted in Med-OFF and improvement of the symptoms was observed with levodopa treatment.
